# Impact of Catheter Ablation on Long-Term Outcomes in Patients With Atrial Fibrillation: A Meta-Analysis

**DOI:** 10.7759/cureus.29202

**Published:** 2022-09-15

**Authors:** Tanveer Ahamad Shaik, Muhammad Haseeb, Sana Faisal, Kinan Obeidat, Osama Salam, Jithin Karedath, Zubair Ahmad Ganaie, Shamsha Hirani

**Affiliations:** 1 Cardiovascular Medicine, University of Louisville School of Medicine, Louisville, USA; 2 Pediatric Medicine, Jackson Park Hospital, llinois, USA; 3 Medical School, Dow University of Health Sciences, Karachi, PAK; 4 Internal Medicine, University of Texas, Austin, USA; 5 General Medicine, James Cook University Hospital, Middlesbrough, GBR; 6 Internal Medicine, Holy Family Red Crescent Medical College, Srinagar, IND; 7 Cardiology, Baqai Hospital, Karachi, PAK

**Keywords:** all-cause mortality, stroke, meta-analysis, atrial fibrillation, catheter ablation

## Abstract

The role of catheter ablation in patients with atrial fibrillation (AF) in enhancing long-term outcomes remains unknown. This meta-analysis aimed to assess the impact of catheter ablation on stroke, all-cause mortality, hospitalization due to heart failure, and major bleeding events in patients with atrial fibrillation. This meta-analysis was conducted in compliance with the Preferred Reporting Items for Systematic Reviews and Meta-Analyses (PRISMA) guidelines. The data search was carried out by two authors independently using online databases including PubMed, EMBASE, and Cochrane library. The primary outcome was a stroke. The secondary outcomes were all-cause mortality, hospitalization for heart failure, and major bleeding events. Total, 10 articles were included in the current meta-analysis encompassing 275392 patients (33291 in the ablation group and 244974 in the non-ablation group). Among all included studies, one study was a randomized control trial, while the remaining other were retrospective cohorts. The current meta-analysis showed that catheter-based AF ablation reduced the risk of stroke (hazard ratio {HR}: 0.61, 95% CI: 0.49-0.77), all-cause mortality (HR: 0.60, 95% CI: 0.51-0.71), and hospitalization for heart failure (HR: 0.57, 95% CI: 0.43-0.76). No significant differences were reported in terms of major bleeding events between patients who received catheter-based AF ablation and patients who did not receive catheter-based AF ablation (HR: 0.96, 95% CI: 0.80-1.14). In the current meta-analysis, catheter-based AF ablation was associated with decreased risk of all-cause mortality, stroke, and hospitalization due to heart failure. However, no significant difference was reported in terms of major bleeding events.

## Introduction and background

Atrial fibrillation (AF) affects 3 to 5 million people in the United States and its prevalence and incidence have been increasing every year [1]. Many patients who develop atrial fibrillation have certain comorbidities like structural heart disease, hypertension, and diabetes [2]. Thus, these patients experience a significant risk of cardiovascular events like heart failure and stroke [3].

Compared to just controlling the ventricular rate, sinus rhythm maintenance (rhythm control), which reflects the normal heart rhythm, may be thought of as the most natural method (rate control) [4]. However, rate control has been shown in major clinical trials to be non-inferior to rhythm control with antiarrhythmic medications (AADs) in terms of preventing death, stroke, and heart failure [5]. The adverse effects and proarrhythmic properties of anti-arrhythmic drugs along with their relatively modest efficacy in preserving sinus rhythm, most certainly account for this [6]. Catheter ablation for atrial fibrillation has grown into an important and effective option for many patients since its initial description in the late 1990s [7]. Several clinical trials have shown that atrial fibrillation ablation maintains sinus rhythm more efficiently as compared to anti-arrhythmic drugs as a second-line and possibly a first-line strategy [8-9]. This improved sinus rhythm is linked with enhanced symptoms [10].

These trials have been debated widely but are not powered enough to show a possible effect on the outcomes like all-cause mortality, hospitalization related to cardiovascular events, and stroke in patients undergoing atrial fibrillation catheter ablation [11] or affected by the crossover between the study groups [12]. On the other hand, findings from retrospective studies have shown the benefits of atrial fibrillation catheter ablation in terms of clinical endpoint reductions.

There is a paucity of data related to the impact of catheter ablation on outcomes like mortality and stroke. To fill this knowledge gap, this meta-analysis was performed to determine the effectiveness of catheter-based AF ablation by evaluating the impacts of catheter ablation on stroke, all-cause mortality, hospitalization due to heart failure, and major bleeding events in patients with atrial fibrillation.

## Review

Methods

This meta-analysis was conducted in compliance with the Preferred Reporting Items for Systematic Reviews and Meta-Analyses (PRISMA) guidelines.

Search Strategy

The data search was carried out by two authors independently using online databases including PubMed, EMBASE and Cochrane library. The electronic search was carried out by combining the following key terms and Medical Subject Headings (MeSH) terms: “atrial fibrillation”, “Catheter ablation”, “Hospitalization”, “death”, and “stroke”. A reference list of all relevant studies was reviewed as well. All references were downloaded into EndNote Version X9 (Thompson ISI ResearchSoft, Philadelphia, Pennsylvania). Duplicates were removed manually and electronically.

Study Selection

Two investigators reviewed the title and abstract of studies independently to determine whether they are eligible for full-text review based on the eligibility criteria. Title and abstract screening were followed by a full-text review of relevant articles. Studies included in the current meta-analysis only if they fulfill the following inclusion criteria (a) compared at least one of the three clinical outcomes (stroke, all-cause mortality, and hospitalization for heart failure), (b) Compared catheter ablation with non-ablated atrial fibrillation patients, (c) follow-up of at least one year, (d) include studies recruited patients exclusively with normal left-ventricular ejection fraction (LVEF) of more than or equal to 40%. The inclusion criteria were not limited to sample size and year of publication. Studies were excluded if they were published in a language other than English. The discrepancy between the two authors was resolved via consensus or discussion with a third author.

Quality Assessment

Risk of bias assessment of randomized control trial (RCT) was done using the Cochrane bias risk assessment tool, while Newcastle‐Ottawa Scale was used for observational studies.

Data Extraction

Data were extracted by two authors independently using a structured data collection form. Data collected included author name, year of publication, sample size, intervention, and follow-up time. The discrepancy between the two authors was resolved via consensus or discussion with a third author.

Outcome Measures

The primary outcome was a stroke. The secondary outcomes were all-cause mortality, hospitalization for heart failure, and major bleeding events. The definitions of the endpoints were taken as reported in the included studies.

Statistical Analysis

Statistical analysis was performed using RevMan version 5.4.0 (The Nordic Cochrane Centre, The Cochrane Collaboration, Copenhagen) and R version 4.1.2 (R Foundation for Statistical Computing, Vienna, Austria). Estimates were pooled using a generic invariance-weighted random or fixed effect model. Outcomes were computed as hazard ratio (HR) with a 95% confidence interval (CI). A p-value of 0.05 was set as significant. A Forest plot was drawn for each outcome representing an individual estimate of each study and pooled estimates. Heterogeneity was assessed utilizing Cochran's Q statistic and I-square. For significant heterogeneity, a p-value<0.1 was considered significant. Heterogeneity was considered low (I^2^<25%), moderate (25-50%) and high (>50%). For publication bias, the Egger test was used. P-value<0.05 was considered significant for the publication bias. To identify the reasons for heterogeneity, meta-regression was also performed to identify the potential association of moderator variables with the primary endpoint (stroke) by taking the age of patients, gender, follow-up time, the prevalence of diabetes mellitus, hypertension, and prevalence of heart failure as moderator variables. A P-value less than 0.05 was considered significant.

Results

**Figure 1:**
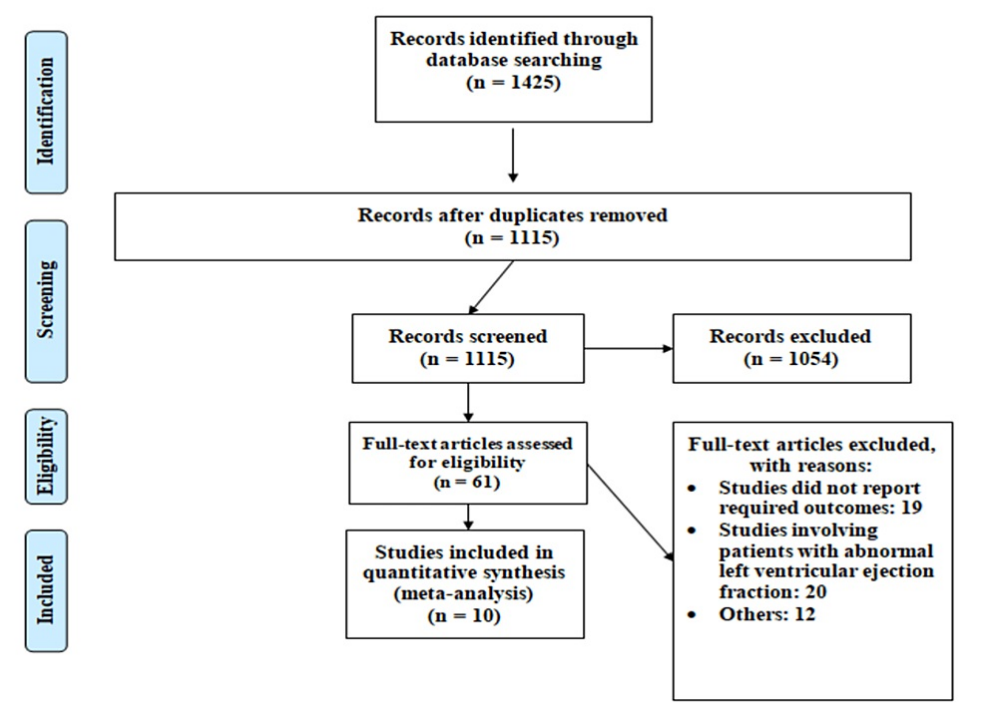
PRISMA flowchart of selection of studies

Figure 1 shows the PRISMA flowchart for the selection of studies. Overall, online searching yielded 1425 studies, of which 310 citations were removed as duplicates. Of the remaining 1115 articles, 1054 studies were excluded after the title and abstract screening. The full text of 61 articles was reviewed for eligibility criteria. Out of 61 articles, 10 articles fulfilled the eligibility criteria and were included in the current meta-analysis encompassing 275392 patients (33291 in the ablation group and 244974 in the non-ablation group).

Table 1 shows the characteristics of all included studies. Among all included studies, one study was a randomized control trial, while the remaining studies were retrospective observational studies. The follow-up period of included studies ranged from 1 year to 4.5 years. In most of the studies, the majority of the participants were males.

**Table 1:** Characteristics of included studies AF: Atrial fibrillation; RCT: Randomized control trial; PSM: Propensity score matching; PSW: Propensity score weighting; SD: Standard deviation

First author	Year	Study type	Population	Groups	Sample Size	Follow-up time	Analysis	Mean age (SD)	Male n(%)
Chang et al. [13]	2014	Retrospective cohort	Patients with AF without prior stroke or heart failure	Ablation	846	3.5 Years	PSM	51.91 (15.30)	599 (70.8)
				Non-ablation	11324			66.98 (12.69)	6719 (59.3)
Ding et al. [14]	2022	Retrospective cohort	Patients with a diagnosis of AF	Ablation	445	3 Years	PSM	63 (9.62)	259 (58.2)
				Non-ablation	25518			71 (10.37)	14068 (55.1)
Friberg et al. [15]	2016	Retrospective cohort	Patients with a diagnosis of AF	Ablation	2496	4.4 Years	PSM	59.97 (10.20)	1892 (75.8)
				Non-ablation	2496			59.55 (12.83)	1879 (76.2)
Joza et al. [16]	2018	Retrospective cohort	Patients with a diagnosis of AF	Ablation	1240	3.5 Years	PSM	67.6 (7.6)	758 (61.1)
				Non-ablation	2427			68.2 (7.6)	1481 (61.0)
Noseworthy et al. [17]	2018	Retrospective cohort	Patients with a diagnosis of AF	Ablation	12032	2.1 Years	PSW	63.7 (11.1)	7881 (65.5)
				Non-ablation	171728			63.7 (11.9)	112482 (65.5)
Packer et al. [12]	2019	RCT	Patients with a diagnosis of AF	Ablation	1108	4 Years	NA	68 (7.41)	695 (62.7)
				Non-ablation	1096			67 (7.41)	690 (63.0)
Reynolds et al. [18]	2012	Retrospective cohort	Patients with a clinical diagnosis of AF	Ablation	801	3 Years	PSM	-	488 (60.9)
				Non-ablation	801			-	501 (62.6)
Saliba et al. [19]	2016	Retrospective cohort	Patients with a diagnosis of AF	Ablation	969	4 Years	PSM	-	613 (63.3)
				Non-ablation	3772			-	2369 (63.7)
Srivatsa et al. [20]	2018	Retrospective cohort	Patients with a diagnosis of AF	Ablation	4169	3.5 Years	PSM	-	3013 (72.3)
				Non-ablation	4169			-	2968 (71.2)
Yang et al. [21]	2020	Retrospective cohort	Patients with a diagnosis of AF	Ablation	9185	3.5 Years	PSW	61 (11.11)	6696 (72.9)
				Non-ablation	18770			62 (12.59)	13402 (71.4)

Table 2 shows the quality assessment of all included retrospective studies. All included retrospective studies have good quality. Table 3 shows the risk of bias assessment of the RCT included in the current meta-analysis. The risk of bias in that RCT is moderate.

**Table 2:** Risk of bias assessment of retrospective studies

Study Id	Selection	Comparibility	Outcome	Overall quality
Chang et al., 2014 [13]	4	1	3	Good
Ding et al., 2022 [14]	4	1	3	Good
Friberg et al., 2016 [15]	4	1	3	Good
Joza et al., 2018 [16]	4	1	3	Good
Noseworthy et al., 2018 [17]	4	2	2	Good
Reynolds et al., 2012 [18]	4	1	3	Good
Saliba et al., 2016 [19]	4	1	3	Good
Srivatsa et al., 2018 [20]	4	1	3	Good
Yang et al., 2020 [21]	4	1	3	Good

**Table 3:** Risk of bias assessment of included RCT

Study Id	Selection bias	Performance bias	Detection bias	Attrition bias	Reporting bias
Packer et al. [12]	Low	High	Low	Low	Low

Comparison of Outcomes Between Ablation and Non-Ablation Groups

**Figure 2:**
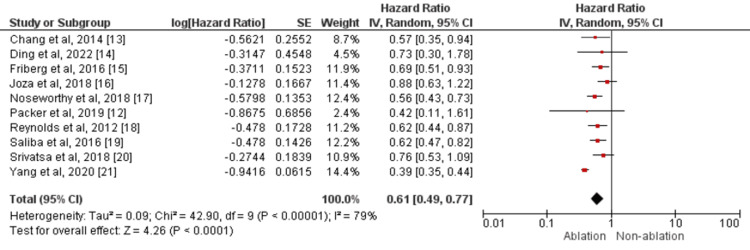
Forest plots comparing ablation vs. no ablation groups in terms of prevention of stroke Sources: References [12-21]

A total of 10 studies compared the stroke between patients who received catheter ablation and patients who did not receive ablation [12,13-21]. By random‐effect model meta‐analysis, catheter-based AF ablation reduced the risk of stroke by 39% (HR: 0.61, 95% CI: 0.49-0.77) compared with patients treated with medical therapy as shown in Figure 2. Heterogeneity was high as shown by its I-square value (I^2^=79%). No publication bias was there for the stroke as the Egger test p-value was 0.36.

**Figure 3:**
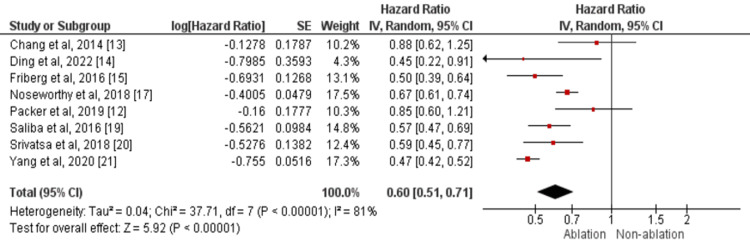
Forest plots comparing ablation vs. no ablation groups in terms of risk of all-cause mortality Sources: References [12-15,17,19-21]

A total of eight studies assessed the all-cause mortality between the study groups [12-15,17,19-21]. The risk of all-cause mortality was significantly lower in patients receiving catheter-based AF ablation compared to patients treated with medical therapy (HR: 0.60, 95% CI: 0.51-0.71) as shown in Figure 3. Significant heterogeneity was there among the study results (I-square=81%). However, no evidence of publication bias was found in the outcome of interest (P-value of Egger test= 0.18).

**Figure 4:**
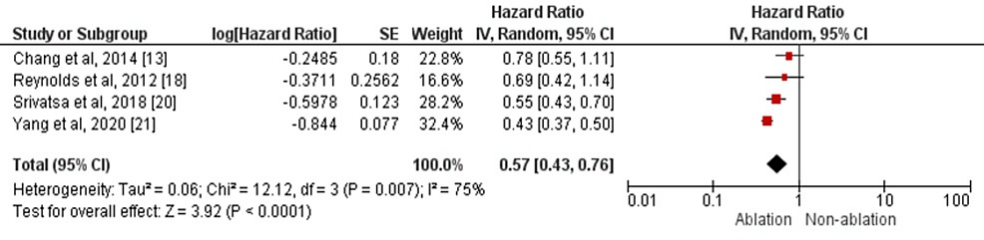
Forest plots comparing ablation vs. no ablation groups in terms of hospitalization for heart failure Sources: References [13,18,20-21]

Hospitalization for heart failure was assessed by four studies [13,18,20-21]. Catheter-based AF ablation decreased the risk of hospitalization for heart failure in patients with atrial fibrillation as compared to its counterparts (HR: 0.57, 95% CI: 0.43-0.76) as shown in Figure 4. Significant heterogeneity was there among the study results (I-square=75%). However, no evidence of publication bias was found in the outcome of interest (P-value of Egger test=0.33).

**Figure 5:**
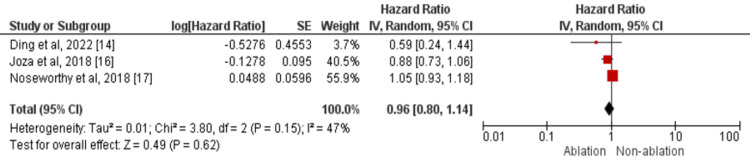
Forest plots comparing ablation vs. no ablation groups in terms of major bleeding events Sources: References [14,16-17]

The risk of major bleeding events was assessed by three studies in the current meta-analysis [14,16-17]. No significant difference was found between the two study groups in terms of major bleeding events (HR: 0.96, 95% CI: 0.80-1.14) as shown in Figure 5. Significant heterogeneity was there among the study results (I-square=47%).

Meta-Regression

The evaluation of potential moderator variables via meta-regression showed that the benefits of catheter ablation for the decrease in all-cause mortality were explained by the mean age of patients and history of cerebrovascular disease and coronary artery disease. For studies with higher age of patients, catheter ablation was associated with decreased benefit in the reduction of all-cause mortality (P-value=0.002), and hypertension (P-value=0.011)

Sensitivity Analysis

Table 4 shows the results of the sensitivity analysis. Findings of sensitivity analysis showed that by analyzing retrospective studies including propensity score matching, findings in terms of stroke and all-cause mortality were similar to overall findings. However, RCT conducted by Packer et al. [12] did not show any significant impact of catheter ablation on stroke and all-cause mortality. 

**Table 4:** Results of sensitivity analysis PSM: Propensity score matching; PSW: Propensity score weighting; RCT: Randomized control trial

Study Type	Stroke	I2	All-cause mortality	I2
PSM [13-16, 18-20]	0.69 (0.60-0.79)	0%	0.59 (0.49-0.71)	46%
PSW [17, 21]	0.46 (0.32-0.65)	54%	0.56 (0.40-0.79)	52%
RCT [12]	0.42 (0.11-1.61)	-	0.85 (0.60-1.21)	-

Discussion

Even though atrial fibrillation catheter ablation has historically been used for improving quality of life and symptom relief, whether it offers survival benefits has been a matter of controversy [22]. The current meta-analysis involving pooled analysis of RCT and retrospective cohort studies provides insights in this regard. In patients with atrial fibrillation, catheter ablation can reduce the risk of all-cause mortality, stroke, and hospitalization. Moreover, in terms of safety, no significant difference is there in major bleeding events between patients who received catheter ablation and patients who received medical therapy.

Currently, atrial fibrillation catheter ablation is an option for rhythm control in patients with atrial fibrillation who remain symptomatic on adequate rate control therapy. On the other hand, the demonstrated improved efficacy and safety of catheter ablation in preserving sinus rhythm raises concerns about a potential positive prognostic impact of the modern rhythm control method, either with catheter ablation only or with a combination of catheter ablation and anti-arrhythmic drugs. RCT included in this meta-analysis [12] used intention to treat analysis and sensitivity analysis showed that no significant difference was reported in stroke and all-cause mortality between catheter ablation and medical therapy. The results of this trial were most likely to be affected by high treatment cross-over and lower mortality rate than expected.

Various studies have shown that catheter ablation is better than medical treatment alone for the prevention of recurrent atrial fibrillation, enhancement in quality of life, and reduction of morbidity [23]. Additionally, it may appear reasonable that successful atrial fibrillation catheter ablation will result in a lower risk of mortality and cerebrovascular-related events compared to antiarrhythmic medication therapy alone (a) because people with atrial fibrillation had significantly lower survival rates than subjects without atrial fibrillation [24] and increased risk of cardiac failure, (b) the burden of atrial fibrillation has been proven to be associated with the risk of stroke [25], (c) ablation is more effective than anti-arrhythmic drugs to reduce the atrial fibrillation burden and maintaining sinus rhythm [24]. Theoretically, successful atrial fibrillation catheter ablation could decrease all-cause mortality by preventing thromboembolic outcomes, cardiovascular mortality, and heart failure decompensation as shown by the Framingham Heart Study [26], so simple by permitting patients to stop their anti-arrhythmic medications.

International guidelines currently encourage reserving atrial fibrillation catheter ablation for patients who have experienced at least one anti-arrhythmic medication therapy failure, while it may be taken into consideration in some patients with early forms of atrial fibrillation or heart failure with low ejection fraction [27-28]. In several countries, health care structures are such that often a delay is observed between the diagnosis of atrial fibrillation and specialist review and subsequent referral for consideration of atrial fibrillation [14]. As a result, the adoption of atrial fibrillation ablation is uncommon [14]. However, increasing evidence shows that atrial fibrillation ablation needs to be considered early [29]. Moreover, it is crucial to emphasize the significance of preserving sinus rhythm [30]. The effectiveness of catheter ablation is decreased with delays in treatment [31]. In order to achieve this goal, methodical advancements are required to make it easier to deliver early atrial ablation to patients who are qualified and may benefit from such treatment. This facilitation supports the need for patient care pathways to be more integrated, including for patients with atrial fibrillation and other chronic cardiac illnesses [32].

Limitations

One of the main limitations of this meta-analysis is linked to its methodology and the heterogeneity between studies. The pooled analysis of all-cause mortality, stroke, and hospitalization was graded for heterogeneity using the I-square test. Considering the methodological variations between observational studies, this was anticipated from the start. Besides this, the majority of the studies included in the current meta-analysis are retrospective in nature that needs to be interpreted cautiously because of their observational nature. Although multivariable analysis and matching using propensity analysis can control confounding variables, residual confounding attributed to unmeasured factors remains a concern.

## Conclusions

In the current meta-analysis, catheter-based AF ablation was associated with decreased risk of all-cause mortality, stroke, and hospitalization due to heart failure. However, no significant difference was reported in terms of major bleeding events. Only observational studies have shown a decrease in the risk of stroke. To determine whether ablation can improve survival in populations and reduce stroke, additional well-powered randomized control trials are required.

## References

[1] Miyasaka Y, Barnes ME, Gersh BJ (2006). Secular trends in incidence of atrial fibrillation in Olmsted County, Minnesota, 1980 to 2000, and implications on the projections for future prevalence. Circulation.

[2] Benjamin EJ, Levy D, Vaziri SM, D'Agostino RB, Belanger AJ, Wolf PA (1994). Independent risk factors for atrial fibrillation in a population-based cohort. The Framingham Heart Study. JAMA.

[3] Krahn AD, Manfreda J, Tate RB, Mathewson FA, Cuddy TE (1995). The natural history of atrial fibrillation: incidence, risk factors, and prognosis in the Manitoba Follow-Up Study. Am J Med.

[4] Wyse DG, Waldo AL, DiMarco JP (2002). A comparison of rate control and rhythm control in patients with atrial fibrillation. N Engl J Med.

[5] Van Gelder IC, Hagens VE, Bosker HA (2002). A comparison of rate control and rhythm control in patients with recurrent persistent atrial fibrillation. N Engl J Med.

[6] Saglietto A, De Ponti R, Di Biase L (2020). Impact of atrial fibrillation catheter ablation on mortality, stroke, and heart failure hospitalizations: a meta-analysis. J Cardiovasc Electrophysiol.

[7] Camm AJ, Lip GY, De Caterina R (2012). 2012 focused update of the ESC Guidelines for the management of atrial fibrillation: an update of the 2010 ESC Guidelines for the management of atrial fibrillation. Developed with the special contribution of the European Heart Rhythm Association. Eur Heart J.

[8] Terasawa T, Balk EM, Chung M, Garlitski AC, Alsheikh-Ali AA, Lau J, Ip S (2009). Systematic review: comparative effectiveness of radiofrequency catheter ablation for atrial fibrillation. Ann Intern Med.

[9] Wilber DJ, Pappone C, Neuzil P (2010). Comparison of antiarrhythmic drug therapy and radiofrequency catheter ablation in patients with paroxysmal atrial fibrillation: a randomized controlled trial. JAMA.

[10] Reynolds MR, Walczak J, White SA, Cohen DJ, Wilber DJ (2010). Improvements in symptoms and quality of life in patients with paroxysmal atrial fibrillation treated with radiofrequency catheter ablation versus antiarrhythmic drugs. Circ Cardiovasc Qual Outcomes.

[11] Khan SU, Rahman H, Talluri S, Kaluski E (2018). The clinical benefits and mortality reduction associated with catheter ablation in subjects with atrial fibrillation: a systematic review and meta-analysis. JACC Clin Electrophysiol.

[12] Packer DL, Mark DB, Robb RA (2019). Effect of catheter ablation vs antiarrhythmic drug therapy on mortality, stroke, bleeding, and cardiac arrest among patients with atrial fibrillation: The CABANA randomized clinical trial. JAMA.

[13] Chang CH, Lin JW, Chiu FC, Caffrey JL, Wu LC, Lai MS (2014). Effect of radiofrequency catheter ablation for atrial fibrillation on morbidity and mortality: a nationwide cohort study and propensity score analysis. Circ Arrhythm Electrophysiol.

[14] Ding WY, Calvert P, Gupta D, Huisman MV, Lip GY (2022). Impact of early ablation of atrial fibrillation on long-term outcomes: results from phase II/III of the GLORIA-AF registry. Clin Res Cardiol.

[15] Friberg L, Tabrizi F, Englund A (2016). Catheter ablation for atrial fibrillation is associated with lower incidence of stroke and death: data from Swedish health registries. Eur Heart J.

[16] Joza J, Samuel M, Jackevicius CA (2018). Long-term risk of stroke and bleeding post-atrial fibrillation ablation. J Cardiovasc Electrophysiol.

[17] Noseworthy PA, Gersh BJ, Kent DM, Piccini JP, Packer DL, Shah ND, Yao X (2019). Atrial fibrillation ablation in practice: assessing CABANA generalizability. Eur Heart J.

[18] Reynolds MR, Gunnarsson CL, Hunter TD, Ladapo JA, March JL, Zhang M, Hao SC (2012). Health outcomes with catheter ablation or antiarrhythmic drug therapy in atrial fibrillation: results of a propensity-matched analysis. Circ Cardiovasc Qual Outcomes.

[19] Saliba W, Schliamser JE, Lavi I, Barnett-Griness O, Gronich N, Rennert G (2017). Catheter ablation of atrial fibrillation is associated with reduced risk of stroke and mortality: a propensity score-matched analysis. Heart Rhythm.

[20] Srivatsa UN, Danielsen B, Amsterdam EA (2018). CAABL-AF (California Study of Ablation for Atrial Fibrillation): mortality and stroke, 2005 to 2013. Circ Arrhythm Electrophysiol.

[21] Yang PS, Sung JH, Jang E (2020). Catheter ablation improves mortality and other outcomes in real-world patients with atrial fibrillation. J Am Heart Assoc.

[22] Barra S, Baran J, Narayanan K (2018). Association of catheter ablation for atrial fibrillation with mortality and stroke: a systematic review and meta-analysis. Int J Cardiol.

[23] Choi AD, Hematpour K, Kukin M, Mittal S, Steinberg JS (2010). Ablation vs medical therapy in the setting of symptomatic atrial fibrillation and left ventricular dysfunction. Congest Heart Fail.

[24] Gentlesk PJ, Sauer WH, Gerstenfeld EP (2007). Reversal of left ventricular dysfunction following ablation of atrial fibrillation. J Cardiovasc Electrophysiol.

[25] Glotzer TV, Daoud EG, Wyse DG (2009). The relationship between daily atrial tachyarrhythmia burden from implantable device diagnostics and stroke risk: the TRENDS study. Circ Arrhythm Electrophysiol.

[26] Benjamin EJ, Wolf PA, D'Agostino RB, Silbershatz H, Kannel WB, Levy D (1998). Impact of atrial fibrillation on the risk of death: the Framingham Heart Study. Circulation.

[27] Hindricks G, Potpara T, Dagres N (2021). 2020 ESC Guidelines for the diagnosis and management of atrial fibrillation developed in collaboration with the European Association for Cardio-Thoracic Surgery (EACTS): The Task Force for the diagnosis and management of atrial fibrillation of the European Society of Cardiology (ESC) Developed with the special contribution of the European Heart Rhythm Association (EHRA) of the ESC. Eur Heart J.

[28] January CT, Wann LS, Alpert JS (2014). 2014 AHA/ACC/HRS guideline for the management of patients with atrial fibrillation: a report of the American College of Cardiology/American Heart Association Task Force on Practice Guidelines and the Heart Rhythm Society. J Am Coll Cardiol.

[29] Nattel S, Harada M (2014). Atrial remodeling and atrial fibrillation: recent advances and translational perspectives. J Am Coll Cardiol.

[30] Purkayastha S, Romero J, Lakkireddy D (2022). Contemporary ablation techniques for atrial fibrillation: an evidence-based analysis. Authorea.

[31] Chew DS, Black-Maier E, Loring Z (2020). Diagnosis-to-ablation time and recurrence of atrial fibrillation following catheter ablation: a systematic review and meta-analysis of observational studies. Circ Arrhythm Electrophysiol.

[32] Field M, Kuduvalli M, Torella F, McKay V, Khalatbari A, Lip GY (2022). Integrated care systems and the aortovascular hub. Thromb Haemost.

